# Using a realist approach to evaluate smoking cessation interventions targeting pregnant women and young people

**DOI:** 10.1186/1472-6963-10-49

**Published:** 2010-02-23

**Authors:** Flora CG Douglas, Denise A Gray, Edwin R van Teijlingen

**Affiliations:** 1 Section of Population Health, School of Medicine and Dentistry, University of Aberdeen, Foresterhill, Aberdeen, UK; 2 School of Health & Social Care, Bournemouth University, Bournemouth, UK

## Abstract

**Background:**

This paper describes a study protocol designed to evaluate a programme of smoking cessation interventions targeting pregnant women and young people living in urban and rural locations in Northeast Scotland. The study design was developed on so-called 'realist' evaluation principles, which are concerned with the implementation of interventions as well as their outcomes.

**Methods/design:**

A two-phased study was designed based on the Theory of Change (TOC) using mixed methods to assess both process and outcome factors. The study was designed with input from the relevant stakeholders. The mixed-methods approach consists of semi-structured interviews with planners, service providers, service users and non-users. These qualitative interviews will be analysed using a thematic framework approach. The quantitative element of the study will include the analysis of routinely collected data and specific project monitoring data, such as data on service engagement, service use, quit rates and changes in smoking status.

**Discussion:**

The process of involving key stakeholders was conducted using logic modelling and TOC tools. Engaging stakeholders, including those responsible for funding, developing and delivering, and those intended to benefit from interventions aimed at them, in their evaluation design, are considered by many to increase the validity and rigour of the subsequent evidence generated. This study is intended to determine not only the components and processes, but also the possible effectiveness of this set of health interventions, and contribute to the evidence base about smoking cessation interventions aimed at priority groups in Scotland. It is also anticipated that this study will contribute to the ongoing debate about the role and challenges of 'realist' evaluation approaches in general, and the utility of logic modelling and TOC approaches in particular, for evaluation of complex health interventions.

## Background

In 2007, a team of researchers from the University of Aberdeen was commissioned by NHS Grampian to conduct an external evaluation of six local smoking cessation pilot projects (SCPPs) targeting pregnant women and young people in the Northeast of Scotland. The SCPPs were awarded Scottish Government funding which had been made available to support the newly introduced ban on smoking in public places [1]. This national funding was distributed to regional health boards who, in turn, allocated the funding to local Community Health Partnerships (CHPs) on the basis of their peer-reviewed intervention proposals. This paper describes a study protocol that has been designed to evaluate those SCPP proposals that received funding.

Interventions of this type are generally complex and dynamic; often evolving in response to local circumstances, target group engagement and other events beyond the control of the implementers [2] which can adversely (or otherwise) affect the impact of the intervention [3]. Pawson and Tilley have advocated the use of evaluation study designs that are capable of dealing with these issues [4].

For those reasons, this protocol and study design based on three core principles: (a) the need to verify the characteristics and quality of the intervention at the outset of the evaluation; (b) assess the intervention fairly against process and outcome indicators that; and are (c) 'ideally' identified and defined through a process of consultation with all stakeholders i.e. funders, intervention planners and implementers, and those intended to benefit from the intervention [5,6]. Therefore, this evaluation will use an integrated process and outcomes study design, using so-called 'realist' principles which are concerned with illuminating the *context *in which an intervention is implemented; the *mechanisms *of that intervention, as well as its outcomes [7,4].

## Smoking - the public health problem

### Pregnant women and young people

#### Prevalence and trends in smoking behaviour

In Scotland, approximately 20.9% of women smoke during the first trimester of pregnancy. Whilst there has been a reduction in the level of smoking in recent years, rates are still significantly higher in women from particular social backgrounds and age groups. Smoking in pregnancy rates for women living in the most deprived areas Scottish Index of Multiple Deprivation 5 (SIMD 5) of Scotland are 33.4% compared to 7.4% for women who live in the least deprived areas (SIMD 1). Rates are also considerably higher in the under 20 and 20-24 age groups, 40% and 31.0% respectively [8].

Although there has been a decline in the smoking rates of young people, levels of smoking among young people, girls in particular, remains high. The Scottish Schools Adolescent Lifestyle and Substance Use Survey (SALSUS) 2006 reported that 4% of 13 year olds; (3% boys and 5% girls) and 15% percent of 15 year olds; (12% boys and 18% girls) were regular smokers [9].

### Regional policy and practice

Government funding was increased to enable the expansion of regional smoking cessation services to support targeted efforts to engage pregnant women, children and young people, particularly those living in deprived areas. The ultimate aim of this work is to improve access to services and reducing smoking rates in these groups [10,11].

#### Local smoking cessation service provision

Current service provision in NHS Grampian (Northeast Scotland) is fairly comprehensive and operates across a diverse geographical area. The Smoking Advice Service (SAS) typically offers its clients an intensive support programme of 1 hour group sessions over a 6-7 week period, led by a trained smoking cessation advisor.

#### Pregnant women

Studies suggest that pregnant women would benefit from sustained cessation support throughout pregnancy and may engage more successfully with a support service if home visits are offered. However there is still some debate concerning the most appropriate person to deliver the service e.g. midwife versus other health professional [12,13].

National guidance on smoking cessation services for pregnant women recommended two key implementation priorities. These include, [1] visiting pregnant women at home if they find it difficult to attend specialist services, and [2], monitoring smoking status and offering smoking cessation advice, encouragement and support throughout the pregnancy and beyond [14].

#### Young people

Existing smoking cessation service provision may not be meeting the preferences and needs of young people resulting in poor service uptake and engagement of young people [15]. Young people tend not to contact formal services for smoking advice; more commonly seeking advice from family or friends [9]. Other evidence suggests that adolescents and young people would prefer a more informal, non-school based service which offers flexible support and guidance [16,17].

Clearly, there is limited evidence about the most effective methods of engaging and supporting these two groups of smokers to quit, particularly for individuals living in disadvantaged circumstances [18-20]. Nevertheless, reducing smoking in these groups remains a public health goal in Scotland.

#### The local interventions

NHS Grampian funded its three regional community health partnerships with the twin aims of:

• Developing and piloting of a modified version of the existing Smoking Advice Service (SAS) to meet the needs of people living in disadvantaged areas;

• Creating supportive environments that would increase the availability of a tailored SAS for young people and pregnant smokers living in disadvantaged circumstances.

The services were at an early stage of development at the outset of the evaluation and details of the interventions were lacking at that point. Consequently, the evaluators were expected to map the components of the interventions in addition to identifying outcomes and outcome measures using a Logic Modelling process.

## Evaluating complex health interventions

Smoking cessation programmes, typically aim to positively change a health behaviour, which in turn, is determined by a diverse range of individual (e.g. cognitive and affective) and environmental (social and cultural) factors. Moreover, such interventions set out to achieve positive outcomes in ever changing social, organisational, economic and political contexts [21-23].

These types of interventions are not easily evaluated by traditional experimental designs [24-27]. Health researchers are increasingly urged to use evaluation designs which not only capture outcomes but which also elucidates implementation processes, and contextual factors (internal and external) that may influence the success (or otherwise) of an intervention. Collectively, these data can provide a clearer link (or otherwise) between the intervention and observed outcomes; thus making any claims of attribution more valid [28,2,4,31,7]. Furthermore, it is important to ensure 'implementation failure' is eliminated as an explanation for the failure of some programmes to reach their desired outcomes [32-34].

It is also crucial to identify relevant process and outcome indicators that accurately reflect the nature and intensity of the intervention under review [32,35]. Ideally, the process of identifying relevant indicators and measures should be done as a participatory and collaborative process with all key stakeholders [36,6,37].

Phased study designs are posited as an evaluation approach that can: (a) map the context, components and complexity of emergent health promotion interventions; and (b) identify and select plausible and realistic process and outcome measures on which to judge the merit or worth of the intervention at a later point in time. The MRC (Medical Research Council) framework for example, recommends - as a first stage - that the 'active ingredients' of an intervention, and its likely impact on health outcomes are identified, before attempting a larger scale intervention study [28].

The Theory of Change (ToC) approach, which has been championed by realist methodologists, [4,3] was developed in the US in an effort to find ways of evaluating processes and outcomes in community-based programmes that were not adequately addressed by existing approaches [37]. The ToC approach is defined as *'a systematic and cumulative study of the links between activities, outcomes and contexts of the initiative' *[38]. In generating a programme ToC, steps are taken to link the original problem; the context in which the programmes operates; the planned activities with its intended medium and longer-term outcomes.

Previous research suggests that evaluators should engage with key stakeholders at the early planning and development stage to identify a programme ToC that will link outcomes and activities and explain *how *and *why *the desired change is expected to come about [21,10]. Proponents of ToC often advocate the use of logic models in this process, as a means of identifying an intervention's inputs and activities and its intended outcomes [39].

### Aims and objectives

The overall aim of this study is to evaluate six SCPPs offering smoking cessation support services; three are aimed at young people and three at pregnant women living in Grampian. As this protocol is based on realist evaluation [4], there is no hypothesis, but the two overarching research questions were: "How did the interventions operate?" and "Did they reach their own aims and objectives?"

The objectives of the evaluation are to establish:

1. the intervention processes;

2. any changes made to the six interventions in response to local conditions;

3. the uptake of the services by the intended target group;

4. the degree to which project services become established and stable;

5. the acceptability of the interventions by intended clients and service providers;

6. to what extent the SCPPs met their short and medium term-objectives;

7. the feasibility of further roll out of promising interventions to other areas.

Given the ambiguity of the proposed interventions, the research team proposed a ToC approach to develop an evaluation framework.

## Methods/Design

The evaluation will proceed in two Phases commencing in April 2007 and ending in June 2010. (See Table 1 for research procedure and timeline).

**Table 1:** Research procedure and timeline

1. Phase One - April 2007 until March 2008
Training and support for Logic Modelling process
Organise training workshop for project staff to introduce evaluation proposal and Logic Modelling concept
Develop, refine and confirm individual project Logic Models with project staff.
Agree end of Phase One Logic Models with project staff
Preparation for data collection
Establish baseline data for intended target groups in all three regions; Aberdeen City, Aberdeenshire and Moray.
Prepare and submit ethics application for Phase Two.
Agree short, medium & longer term outcome indicators with project staff
Agree monitoring and reporting of routine and project monitoring data with project staff
Develop draft interview schedules for Phase Two
Formative feedback
Review NICE recommendations on smoking cessation services and feedback to project staff
Prepare and submit final version of Phase One report
Host end Phase 1 feedback sessions for all project staff
2. Phase Two - April 2008 until June 2010
Training and Support for Logic Modelling process
Meet project staff to review ongoing relevance and validity of their project Logic Models
Finalise Logic Models with project staff
Preparation for data collection
Set up database for routine and project monitoring data
Refine and agree final interview schedules
Data collection
Set up and conduct interviews with service users, non-service users and project staff (service providers)
Data analysis
Transcribe interviews; code and analyse
Routine and project monitoring outcome data-file cleaning & validation
Routine and project monitoring data entry and analysis
Publication and dissemination
Prepare and submit interim Phase Two report
Prepare and submit draft final report
Write up and submit final report
Prepare for & deliver final feedback event for all staff
Prepare evaluation findings summary paper

### Phase One (Exploratory Stage)

The main focus of Phase One was to address evaluation objectives 1 and 2. Researchers worked in close consultation with relevant stakeholders through a series of group meetings and individual discussions to (a) establish individual project baseline data; (b) map and understand the nature of each intervention's processes; (c) identify and define appropriate short, medium and long term outcome measures; and (d) identify any known external or internal factors that might influence implementation and outcomes. This understanding was developed using a logic modelling process, [31,39] which is a technique which helps practitioners, health planners and evaluators to identify the things that need to be done within an intervention to help identify short and medium-term impact and longer term outcomes. The strength of logic modelling is that it links the desired impacts and outputs to the actual activities (processes) of the intervention, to ensure that these activities help contribute towards achieving the (measurable) final outputs. In this study, it was intended that the logic modelling process would produce six individual project logic models and their associated outcome indicators at the end of Phase One.

### Phase Two (Explanatory stage)

Phase Two will focus on evaluation objectives 3 to 7. Mixed-research methods will be used for this part of the study [40] to consider: (a) the extent to which the SCPPs are implemented as intended; (b) the extent they reach their target groups; (c) the acceptability of the SCPPs from the perspective of the service providers and target populations, and their experiences of service delivery and use (or non-use); (d) whether (or not) they are able to meet the short, medium and long-term objectives identified in Phase One.

#### Study Participants

Across all six SCPPs, a purposive sample of approximately 40 managers and health care professionals who were involved in planning or delivering the interventions will be invited to participate in semi-structured face-to-face interviews. Approximately 30 young people and 30 pregnant women who have engaged with the SCPPs will also be interviewed. An additional sample of five young people and five pregnant women who have not fully engaged with the SCPPs will also be invited to take part in the study. To become eligible for the study, all service or non- service users must have registered to receive smoking cessation advice and support (Table 2). Young people are defined as being aged between 11 to 18 years attending secondary school.

**Table 2:** Study inclusion criteria

Project staff	Service users	Non-service users
Involved in either planning or delivering smoking cessation intervention	Registered with SAS and receiving cessation support	Registered with SAS but not receiving cessation support
	Being female, pregnant and a smoker	Residing in target area
	Being a young and a smoker	Consenting to interview
	Residing in target area	
	Consenting to interview	

#### Data collection

Details of every individual who registers with the SCPPs are recorded (with consent) by SCPP staff as part of the Minimum Dataset (MDS) of core data required for anonymous national reporting of clients who access Scottish NHS Board specialist smoking cessation services [41]. Additional, anonymised project specific monitoring data is also collected by SCPP staff (with consent from service users) for analysis by the evaluation team. As all six interventions are each fairly small scale projects and the collection of the MDS is compulsory, it was decided to also collect the additional project specific data from of all service users; hence no sample size calculation was needed for this total sample.

This dataset collates information about those who engage with the services; set a quit date; quit smoking at 4 weeks; and their self-reported smoking behaviour at follow-up points of 3 and 12 months. Individual projects are collecting additional data related to aspects of engagement their target, e.g. how many people expressed an interest in their project over time, methods used to contact prospective clients. A combined anonymised datafile containing the SAS and the project engagement information will then be sent to the evaluation team for quantitative analysis.

Semi-structured interviews will be conducted with individual service users, non-users and service providers using interview guides that will be derived from the evaluation objectives listed in Table 3 below.

**Table 3:** Phase Two Semi-structured interview - evaluation objectives

Project staff	Service users	Non-service users
Experience of service delivery	Experiences of service use	Views and experiences about the barriers to uptake
Barriers or challenges to implementation	Views about the acceptability of the service	
Changes made to services to encourage use or to address unforeseen events or outcomes	Experiences of attempts to quit based on support given by the services	
Assessment of the acceptability and stability of their projects		
Effectiveness in addressing its aims and objectives		

#### Data management and analysis

A descriptive statistical analysis will be conducted of routine SAS and project monitoring data using SPSS (version 16). The qualitative data will be analysed using thematic framework analysis [42] incorporating aspects of grounded theory [43,44]. The latter will include reading and re-reading the transcripts of the qualitative interviews to distil the key themes. NVivo (version 7) and Excel software will be used to manage and analyse the data. Using quantitative methods in conjunction with qualitative approaches can help in triangulation of data. [45,45]

#### Ethical considerations

This study has been approved by the regional research ethics committee. All the participants will be invited to opt-in to the study and sign an informed consent prior to enrolment in the study. The preferences of study participants will be considered by offering them a choice of a face-to-face or telephone interview at a place and time at their convenience. All information sheets and consent forms are designed following the guidelines published by the NHS National Patient Safety Agency (2007) [46].

## Discussion

Our study team were commissioned to evaluate a diverse range of pilot projects; which were being implemented with varying levels of funding and resources, using different intervention strategies. Increasing calls for health researchers to provide more comprehensive accounts of interventions under review, coupled with the demand for evidence-based policy and practice, has led to a greater expectation that evaluations will illuminate what works (or not), with which target groups, and why [24,4].

Therefore, we argue that the phased, realist evaluation approach described in this paper, will not only provide more insight into the precise nature of the interventions implemented in this case, but also help link the activities more closely to the final outcomes (if any). Policy makers and health professionals often come under pressure from both politicians and the media, to develop new ways of tackling long-standing health issues and to demonstrate their effectiveness (or otherwise) within relatively short timeframes. This presents an enormous challenge, given that complex health interventions take time to implement and establish before they become fully operational, and show any detectable impact on mortality or morbidity figures.

This study aims to provide the evaluators, and the SCPP staff with the means to ascertain and map the components of their interventions, and identify plausible project outcome measures that fairly reflect their nature and intensity. There is also potential to provide all SCPP stakeholders with an earlier indication of the likely effectiveness of these interventions, and more evidence about the degree to which the intervention is associated with the observed outcomes, because of the use of logic modelling.

## Competing interests

The authors declare that they have no competing interests.

## Authors' contributions

FD conceived of the study, participated in its design and drafting of the manuscript. DG participated in study design, its coordination and drafting of the manuscript. EvT participated in the study design and drafting of the manuscript. All authors read and approved the final manuscript and are willing to take the responsibility for all content.

## Pre-publication history

The pre-publication history for this paper can be accessed here:

http://www.biomedcentral.com/1472-6963/10/49/prepub
